# Enhanced Antibacterial, Anti-Inflammatory, and Antibiofilm Activities of Tryptophan-Substituted Peptides Derived from Cecropin A-Melittin Hybrid Peptide BP100

**DOI:** 10.3390/molecules29225231

**Published:** 2024-11-05

**Authors:** Sukumar Dinesh Kumar, Eun Young Kim, Naveen Kumar Radhakrishnan, Jeong Kyu Bang, Sungtae Yang, Song Yub Shin

**Affiliations:** 1 Department of Cellular & Molecular Medicine, School of Medicine, Chosun University, Gwangju 61452, Republic of Korea; sdkumarphd@gmail.com (S.D.K.); lovetive@naver.com (E.Y.K.); 2 Graduate School of Biomedical Science, Chosun University, Gwangju 61452, Republic of Korea; naveens4596@gmail.com; 3 Division of Magnetic Resonance, Korea Basic Science Institute (KBSI), Ochang 28119, Republic of Korea; bangjk@kbsi.re.kr; 4 Department of Microbiology, School of Medicine, Chosun University, Gwangju 61452, Republic of Korea; 5 Institute of Well-Aging Medicare & CSU G-LAMP Project Group, Chosun University, Gwangju 61452, Republic of Korea

**Keywords:** antimicrobial peptides, BP100 analogs, tryptophan substitution, antimicrobial activity, anti-inflammatory activity, antibiofilm activity, multidrug resistance

## Abstract

The emergence of multidrug-resistant pathogens necessitates the development of novel antimicrobial agents. BP100, a short α-helical antimicrobial peptide (AMP) derived from cecropin A and melittin, has shown promise as a potential therapeutic. To enhance its efficacy, we designed and synthesized 16 tryptophan-substituted BP100 analogs based on helical wheel projections. Among these, BP5, BP6, BP8, BP11, and BP13 exhibited 1.5- to 5.5-fold higher antibacterial activity and improved cell selectivity compared to BP100. These analogs demonstrated superior efficacy in suppressing pro-inflammatory cytokine release in LPS-stimulated RAW 264.7 cells and eradicating preformed biofilms of multidrug-resistant *Pseudomonas aeruginosa* (MDRPA). Additionally, these analogs showed greater resistance to physiological salts and serum compared to BP100. Mechanistic studies revealed that BP100 and its analogs exert their antibacterial effects through membrane disruption, depolarization, and permeabilization. Notably, these analogs showed synergistic antimicrobial activity with ciprofloxacin against MDRPA. Our findings suggest that these tryptophan-substituted BP100 analogs represent promising candidates for combating multidrug-resistant bacterial infections, offering a multifaceted approach through their antibacterial, anti-inflammatory, and antibiofilm activities.

## 1. Introduction

The rapid emergence and dissemination of multidrug-resistant bacteria pose a significant threat to global public health, underscoring the urgent need for novel antimicrobial agents. Antimicrobial peptides (AMPs), integral components of the eukaryotic innate immune system, have garnered considerable attention as potential therapeutic alternatives due to their broad-spectrum antibacterial activity and low propensity for inducing drug resistance [1]. Furthermore, several AMPs have exhibited antibiofilm activity. Moreover, certain AMPs exhibit antibiofilm properties capable of inhibiting bacterial cell adhesion to surfaces, eradicating cells within the biofilm matrix, and inducing biofilm disaggregation through interactions with extracellular matrix components [2]. The multi-target mechanism of AMPs renders the development of bacterial resistance, as observed with conventional antibiotics, highly improbable.

In addition to their antimicrobial properties, some AMPs demonstrate anti-inflammatory activities by inhibiting the production of pro-inflammatory cytokines induced by endotoxins [3]. This anti-inflammatory response typically occurs through direct binding to endotoxins, such as lipopolysaccharide (LPS) [3,4]. Consequently, AMPs act as scavengers, forming complexes with endotoxins that prevent binding to their receptors (e.g., Toll-like receptor-4). The cationic and amphipathic nature of AMPs appears to be responsible for their affinity for both endotoxins and endotoxin receptors [4,5]. This unique combination of properties makes AMPs highly attractive potential therapeutic agents.

BP100 (KKLFKKILKYL-NH_2_), a short hybrid α-helical AMP designed through the hybridization of cecropin A and melittin, has demonstrated high selectivity toward Gram-negative bacteria [6]. Its low minimal inhibitory concentrations, minimal cytotoxicity, high therapeutic index, and low susceptibility to degradation make BP100 an attractive scaffold for drug development [6].

Certain AMPs, such as indolicidin and tritrpticin, are notably rich in tryptophan (Trp). Trp-rich AMPs exhibit potent activity against bacteria, fungi, viruses, protozoan pathogens, and cancer cells [7,8]. These peptides can deeply penetrate biological membranes due to Trp’s unique chemical properties, which enhance diverse cell-death mechanisms [7,8]. Trp is known for its strong affinity to the interfacial region of biological membranes, facilitating interactions between Trp-containing peptides and membrane surfaces [8]. Studies have demonstrated that substituting one or two Trp residues in α-helical AMPs enhances their antimicrobial activity without significantly increasing their hemolytic effects [9,10]. Certain Trp-containing AMPs have been shown to disrupt quorum sensing and biofilm development in multidrug-resistant *Pseudomonas aeruginosa* [11]. Furthermore, some Trp-substituting AMPs can inhibit the LPS-induced inflammatory response due to their strong binding to LPS [12,13].

Adding an extra Trp to the N-terminus of BP100, W-BP100 significantly enhanced its antibacterial activity against Gram-positive species compared to BP100, while both peptides showed similar effectiveness against Gram-negative bacteria [14]. RW-BP100, with a Trp substitution near the C-terminus of BP100, was notably more effective against Gram-positive bacteria than BP100 [15]. In our recent study, BP100-W, where Trp replaced Leu at position 3 of BP100, showed a 1.7-fold increase in selectivity for bacterial cells and exhibited greater potential in antibiofilm and anti-inflammatory activities compared to BP100 [16].

The present study aimed to develop short α-helical multi-functional AMPs with antibacterial, anti-inflammatory, and antibiofilm activities while minimizing hemolytic activity. We designed BP100 analogs with one, two, and three tryptophan substitutions guided by α-helical wheel projections. The antibacterial efficacy of these peptides was evaluated against various bacteria, including drug-resistant strains, by determining their minimum inhibitory concentrations (MIC). To assess peptide toxicity, we examined their hemolytic activity in sheep erythrocytes and calculated the therapeutic index (TI) to determine cell selectivity. The antibiofilm capabilities of BP100 and its analogs were also assessed, particularly their effectiveness in preventing biofilm formation and eliminating established biofilms of multidrug-resistant *Pseudomonas aeruginosa* (MDRPA). Additionally, we investigated whether BP100 and its analogs could inhibit the production of inflammatory markers (TNF-α and IL-6) in lipopolysaccharide (LPS)-stimulated mouse macrophage RAW 264.7 cells. Consequently, several analogs of BP100, including BP5, BP6, BP8, BP11, and BP13, demonstrated improved cell selectivity, along with enhanced antibiofilm and anti-inflammatory properties compared to the original BP100.

To evaluate the clinical potential of the selected BP100 analogs as antimicrobial agents, we studied their resistance to physiological salts and human serum. Furthermore, we investigated the antibacterial mechanisms of these analogs using techniques such as membrane depolarization and permeabilization of the outer and inner bacterial membranes. We also examined the synergistic effects of combining the selected BP100 analogs with ciprofloxacin against MDRPA to determine their potential as adjuncts in combination drug therapies. Our findings provide a foundation for the development of potent antibacterial agents that offer comprehensive therapeutic benefits while minimizing risks to mammalian cells.

## 2. Results and Discussion

### 2.1. Peptide Design and Characterization

**Figure 1:**
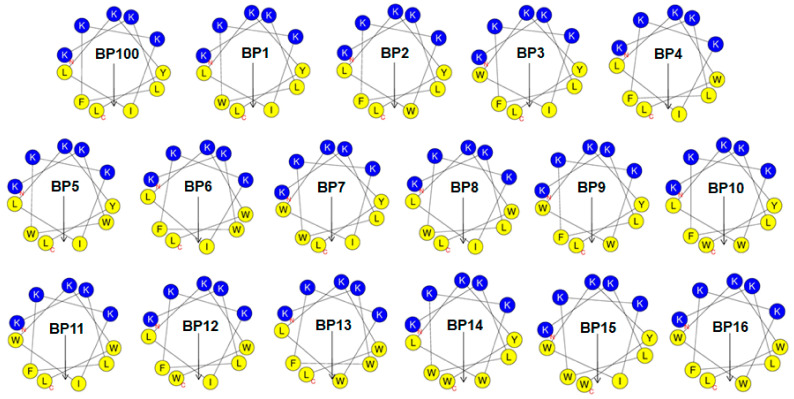
The helical wheel projections of BP100 and its analogs are depicted with color-coded residues. Non-polar hydrophobic residues are shown in yellow, and polar basic residues are shown in dark blue. The hydrophobic moment is represented by a black arrow on the helical wheel.

The α-helical wheel projections for BP100 and its analogs were generated using HeliQuest online tool (https://heliquest.ipmc.cnrs.fr, accessed on 17 September 2023). As illustrated in Figure 1, BP100 displays a distinct amphipathic character, with hydrophilic amino acid residues situated on one face and hydrophobic residues aligned on the opposite face. To maintain the +5 positive net charge, we retained the five original Lys residues on the hydrophilic face of BP100. We then synthesized a series of Trp-substituted BP100 analogs, systematically replacing hydrophobic residues on the hydrophobic face with one, two, or three Trp residues.

The synthesized peptides were designated as follows: one-Trp-substituted analogs (BP1, BP2, BP3, and BP4), two-Trp-substituted analogs (BP5, BP6, BP7, BP8, BP9, BP10, BP11, and BP12), and three-Trp-substituted analogs (BP13, BP14, BP15, and BP16). The theoretical molecular weights of the synthetic peptides were confirmed using matrix-assisted laser desorption ionization-time-of-flight mass spectrometry (MALDI-TOF-MS) (Figure S1). The calculated and measured peptide weights were consistent, confirming the accuracy of the synthesis (Table 1).

### 2.2. Antibacterial Activity and Cell Selectivity

The antibacterial efficacy of the synthesized peptides was evaluated by determining the minimal inhibitory concentrations (MICs) against six standard bacterial strains and five drug-resistant bacterial strains using the Clinical and Laboratory Standards Institute (CLSI) microdilution method [17,18]. Melittin served as the positive control. As presented in Table 2, BP100 and its analogs exhibited broad-spectrum antibacterial activity, with geometric mean (GM) MICs ranging from 4.1 to 32.7 μM. Among the BP100 analogs, ten derivatives (BP2, BP4, BP5, BP6, BP7, BP8, BP11, BP13, BP14, and BP15) demonstrated antibacterial activity approximately 1.2- to 5.5-fold higher than that of the parent peptide BP100. Notably, BP5 (GM: 4.1 μM) displayed the most potent antibacterial activity, being approximately six times more active than melittin (GM = 24.0 μM).

To assess toxicity in mammalian cells, we evaluated the hemolytic activity of the peptides on sheep red blood cells (sRBCs). Remarkably, even at the highest peptide concentration tested (256 μM), BP100 and its analogs exhibited no detectable hemolysis. The therapeutic index (TI), a measure commonly employed to evaluate the cell selectivity of antibacterial agents, was calculated as the ratio of the concentration causing 10% hemolysis of sRBCs (HC_10_) to the GM [19,20,21,22]. As indicated in Table 2, ten analogs (BP2, BP4, BP5, BP6, BP7, BP8, BP11, BP13, BP14, and BP15) demonstrated approximately 1.2- to 5.5-fold higher cell selectivity than BP100. Notably, BP5 exhibited the highest TI (124.9), representing a 5.5-fold improvement over that of BP100 (TI = 22.7).

### 2.3. Anti-Inflammatory Activity

Lipopolysaccharides (LPS), natural ligands for Toll-like receptors (TLRs) on macrophages, are components of the cell wall of Gram-negative bacteria [23]. These molecules can activate macrophages to secrete pro-inflammatory mediators, potentially leading to deleterious inflammatory responses and organ damage. Therefore, we investigated the effect of BP100 and its analogs on the inflammatory response in LPS-induced RAW 264.7 murine macrophages. RAW 264.7 cells were stimulated with LPS from *Escherichia coli* O111:B4 and subsequently treated with 2 μM of each peptide for 24 h. LL-37 served as a positive control. Following incubation, pro-inflammatory cytokines, including tumor necrosis factor-α (TNF-α) and interleukin-6 (IL-6), were quantified using an enzyme-linked immunosorbent assay (ELISA).

**Figure 2:**
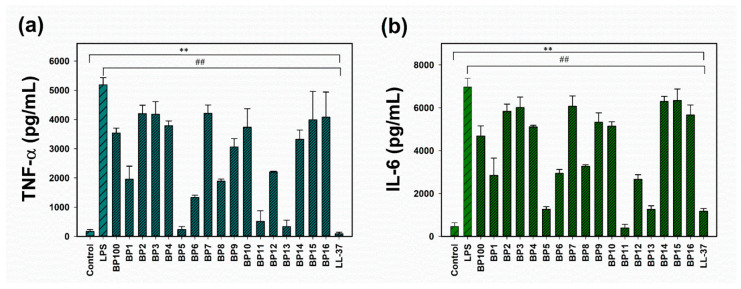
Effects of BP100 and its analogs on the release of pro-inflammatory cytokines from lipopolysaccharide (LPS)-stimulated RAW264.7 macrophage cells. (**a**) Tumor necrosis factor-α (TNF-α) release. (**b**) Interleukin-6 (IL-6) release. Peptides were administered at a concentration of 2 μM. Data represent the mean ± standard error of the mean (SEM) from at least three independent experiments. Statistical analysis was performed using one-way analysis of variance (ANOVA) followed by Duncan’s test. Compared to control: ** *p* < 0.01. Compared to LPS: ^##^ *p* < 0.01.

Prior to assessing anti-inflammatory activity, the biocompatibility of BP100 and its analogs was evaluated on untreated RAW 264.7 cells using an MTT assay. The results demonstrated that BP100 and its analogs were non-toxic to RAW 264.7 cells at concentrations up to 4 μM (Figure S2). Notably, at a non-toxic concentration of 2 μM, several BP100 analogs (BP1, BP5, BP6, BP8, BP11, BP12, and BP13) more effectively suppressed the release of TNF-α and IL-6 in LPS-stimulated RAW 264.7 cells compared to BP100 (Figure 2). These findings suggest that BP100 and the aforementioned analogs (BP1, BP5, BP6, BP8, BP11, BP12, and BP13) are potent anti-inflammatory agents, exhibiting effectiveness comparable to that of the control anti-inflammatory peptide, LL-37 [19].

### 2.4. Antibiofilm Activity

**Figure 3:**
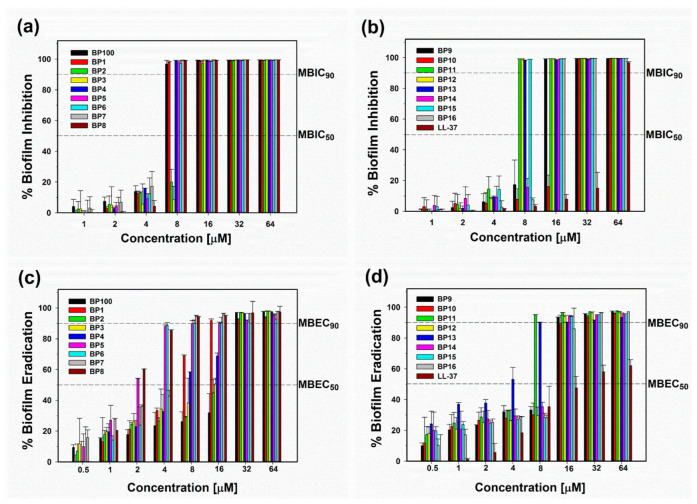
Antibiofilm activity of BP100 and its analogs against multidrug-resistant *Pseudomonas aeruginosa* (MDRPA). (**a**,**b**) Inhibitory activity against biofilm formation. (**c**,**d**) Eradication activity of preformed MDRPA biofilms. Dotted lines indicate 50% inhibition (MBIC_50_), 90% inhibition (MBIC_90_), 50% eradication (MBEC_50_), and 90% eradication (MBEC_90_) concentrations. Values represent the mean ± standard error of the mean (SEM) from three independent experiments.

Bacterial biofilms, comprising communities of bacterial cells encased in a matrix of polysaccharides and nucleic acids, present a significant challenge for conventional antibiotics due to the protective nature of this matrix [24]. Biofilms can enhance bacterial resistance to antibiotics by up to 1000-fold compared to planktonic bacteria [24]. To address this issue, there is an urgent need to discover and develop novel alternative therapeutic agents capable of effectively combating biofilm-forming bacteria. Antimicrobial peptides (AMPs), naturally occurring molecules with bactericidal properties, play a crucial role in this context, although only a subset of AMPs demonstrates the ability to prevent or remove biofilms [25]. Our research specifically focused on evaluating the impact of BP100 and its analogs on biofilm formation and eradication using multidrug-resistant *Pseudomonas aeruginosa* (MDRPA), a common pathogen responsible for biofilm-mediated nosocomial infections. The adhered biomass was quantified using crystal violet staining, and the minimum biofilm inhibitory concentration (MBIC_90_) was determined as the lowest concentration required to inhibit 90% of biofilm growth relative to the positive control. BP100 and its analogs exhibited remarkable antibiofilm activity, showing 2- to 8-fold higher biofilm inhibitory effects compared to LL-37 (Figure 3a,b and Table 3). Human cathelicidin LL-37 is well-documented for its potent antibiofilm properties [26]. The superior activity of BP100 and its analogs against bacterial biofilm formation underscores their potential as antibiofilm agents.

While preventing biofilm formation on surfaces is crucial, treating established biofilm infections presents a significant challenge due to the extracellular polymeric substance (EPS) matrix hindering antibiotic penetration and the high cell density within mature biofilms. To address this challenge, we evaluated the ability of BP100 and its analogs to eradicate established MDRPA biofilms over a 24 h period. The minimum biofilm eradication concentration (MBEC_90_) was determined using crystal violet staining as the lowest concentration that reduced biomass by more than 90%. Among these peptides, BP5, BP6, BP7, BP8, BP9, BP11, and BP13 eradicated preformed MDRPA biofilms by more than 90% at a concentration of 8 μM (Figure 3c,d and Table 3). In conclusion, our results demonstrate that BP1, BP5, BP6, BP7, BP8, BP11, and BP13 exhibit potent inhibitory and eradicative activities against MDRPA biofilms.

Notably, five analogs (BP5, BP6, BP8, BP11, and BP13) of BP100 demonstrated enhanced antibacterial, anti-inflammatory, and antibiofilm activities, surpassing the original BP100 and warranting further investigation.

### 2.5. Salt and Serum Stability

To comprehensively evaluate the therapeutic potential of BP100 and the five selected analogs, we assessed their capacity to maintain antibacterial activity in the presence of physiological salts and serum (Table 4). Antibacterial assays were conducted in an environment supplemented with either 10% fresh human serum or physiological salts. BP100 exhibited a 4-fold decrease in activity against both *Escherichia coli* (KCTC 1682) and *Staphylococcus aureus* (KCTC 1621) when exposed to physiological salts or 10% human serum. In contrast, all BP100 analogs either maintained their activity or demonstrated only a 2-fold reduction in efficacy under these conditions. These findings suggest that the selected analogs possess marginally higher resistance to serum and physiological salts compared to the parent peptide, BP100. This enhanced stability in physiologically relevant conditions may have important implications for the potential therapeutic applications of these peptide analogs.

### 2.6. Secondary Structures of BP100 and Selected Analogs

**Figure 4:**
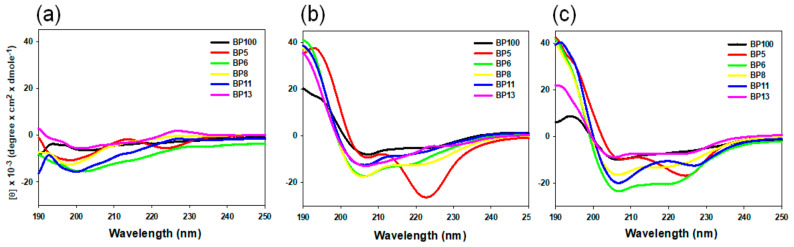
Circular dichroism (CD) spectra of BP100 and its analogs in various environments. (**a**) With 10 mM of sodium phosphate buffer. (**b**) With 50% trifluoroethanol (TFE). (**c**) With 30 mM of sodium dodecyl sulfate (SDS). The mean residue ellipticity was plotted against wavelength. Each spectrum represents the average of three independent scans.

The secondary structures of BP100 and the five selected analogs were investigated in PBS and in membrane-mimicking solvents (50% TFE in PBS and 30 mM of SDS in PBS) using CD spectroscopy (Figure 4). All peptides adopted a mostly random-coil structure in PBS, as demonstrated by the broad minimum peak at 200 nm. In a negative electric environment (30 mM of SDS) and a hydrophobic environment (50% TFE), except BP5, all peptides formed a stable α-helical conformation with two minimum signals around 208 nm and 222 nm and a maximum signal at 192 nm.

### 2.7. Antibacterial Mechanism Studies

To elucidate the antibacterial mechanism of BP100 and the five selected analogs, we employed assays involving membrane depolarization, N-phenyl-1-naphthylamine (NPN) uptake, and propidium iodide (PI) uptake. In these assays, melittin served as the control antimicrobial peptide (AMP) targeting the membrane, while buforin-2 was utilized as the control AMP targeting intracellular components.

#### 2.7.1. Membrane Depolarization

**Figure 5:**
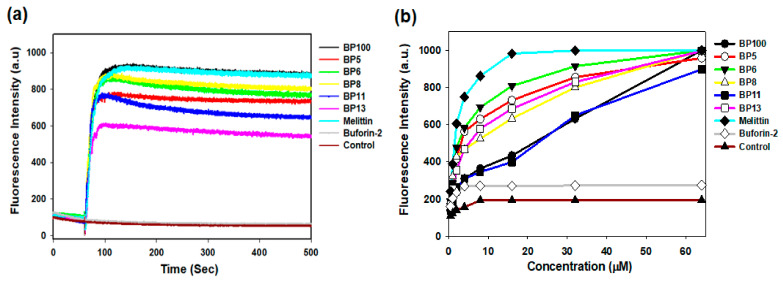
Membrane interaction studies of BP100 and its analogs. (**a**) Time-dependent cytoplasmic membrane depolarization of *Staphylococcus aureus* (KCTC 1621) treated with peptides at 1× MIC (BP100, BP8, BP12, and BP13: 8 μM, BP5 and BP6: 4 μM, Buforin-2: 32 μM), as assessed by the release of the membrane potential-sensitive dye 3,3′-dipropylthiadicarbocyanine iodide (diSC_3_-5). Control experiments conducted in the absence of bacterial cells showed no direct peptide–dye interactions, confirming that fluorescence changes were specifically due to membrane depolarization. (**b**) Outer membrane permeabilization of *Escherichia coli* (KCTC 1682) in the presence of different peptide concentrations, as measured by 1-N-phenylnaphthylamine (NPN) uptake.

Membrane depolarization is a critical event leading to bacterial cell death, primarily by disrupting DNA, RNA, and protein synthesis. We investigated the depolarization effects of BP100 and its analogs using the membrane potential-sensitive dye, 3,3′-dipropylthiadicarbocyanine (diSC_3_-5) [27,28,29]. In the polarized state, diSC_3_-5 accumulates within the bacterial cytoplasmic membrane, resulting in quenched fluorescence. However, depolarization triggers the release of diSC_3_-5 into the external buffer, evidenced by increased fluorescence intensity. We observed that BP100 and its selected analogs, at a concentration of 8 μM, dissipated the membrane potential of the cytoplasmic membrane (CM) in *Staphylococcus aureus* (KCTC 1621) (Figure 5a). This led to a marked and sustained fluorescence increase within 500 s, indicative of CM depolarization, a pattern similar to that caused by melittin.

#### 2.7.2. Outer Membrane Permeability

The outer membrane (OM), characterized by the presence of lipopolysaccharides (LPS), distinguishes Gram-negative bacteria and serves as a formidable barrier against various antimicrobial agents. We utilized *Escherichia coli* (KCTC 1682) as a representative model to investigate how BP100 and its selected analogs influence OM permeability. Changes in permeability were measured using the N-phenyl-1-naphthylamine (NPN) assay, which quantifies fluorescence intensity [27,30]. Under normal conditions, the integrity of the OM prevents NPN from entering the cell. However, if the membrane integrity is compromised, NPN shifts to the cell’s hydrophobic environment, triggering an increase in fluorescence emission. Similar to melittin, we observed a correlation between the concentration of BP100 and its selected analogs and the uptake of NPN; notably, a significant uptake of approximately 100% occurred at a peptide concentration of 32 μM (Figure 5b). These findings indicate that BP100 and its selected analogs can perturb the OM of *E. coli* in a dose-dependent manner, suggesting a cumulative effect on membrane disruption.

#### 2.7.3. Inner Membrane Permeabilization

**Figure 6:**
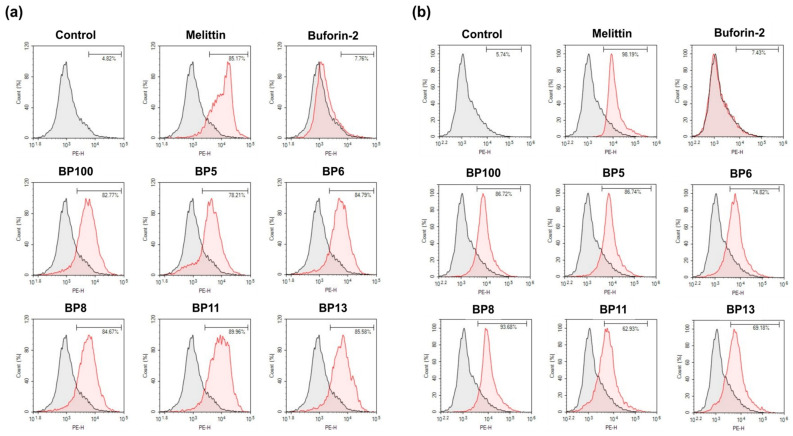
Assessment of bacterial membrane integrity using flow cytometry. (**a**) *Escherichia coli* (KCTC 1682). (**b**) *Staphylococcus aureus* (KCTC 1621). Mid-logarithmic phase bacterial cultures were treated with 1× MIC of peptides, and cellular fluorescence was observed using a FACS scan flow cytometer. Membrane integrity damage was assessed by an increase in fluorescent intensity of propidium iodide (PI, 10 μg/mL) after incubation at 37 °C for 1 h. Control samples were processed without peptide treatment. This analysis provides quantitative data on the membrane-disrupting capabilities of the peptides against both Gram-negative and Gram-positive bacteria.

The permeabilization of the bacterial inner membrane (IM) by BP100 and its analogs was evaluated using propidium iodide (PI). PI, a membrane-impermeant red fluorescent dye that binds to nucleic acids, causes a marked increase in fluorescence intensity by as much as 20–30 times when bound to DNA, compared to its presence in aqueous solutions [27,31]. The peptide-induced disruption of the IM allows PI to enter the cells and bind to DNA, resulting in the bacteria emitting red fluorescence. As shown in Figure 6, unlike buforin-2, BP100 and its selected analogs, similar to melittin, caused substantial cell membrane damage. This is evident in the elevated percentages of PI-positive *E. coli* (KCTC 1682) (ranging from 78.21% to 89.96%) and *S. aureus* (KCTC 1621) (ranging from 62.93% to 93.68%). These findings confirm that BP100 and its selected analogs exert their antibacterial effects by depolarizing, disrupting, and permeabilizing bacterial cell membranes.

### 2.8. Synergistic Antibacterial Effect with Ciprofloxacin

Multidrug-resistant pathogens pose a significant challenge to both human and animal health. The steady decline in the discovery of new antibiotics has exacerbated the difficulty in developing effective therapies for infection control. Consequently, there is an urgent need for alternative strategies that can combat pathogens without inducing drug resistance. One such strategy involves the combination of different antimicrobial agents. The effects of antimicrobial peptides (AMPs) combined with conventional antibiotics often surpass those of individual drugs. Therefore, the development of AMPs that exhibit synergistic effects with antibiotics against multidrug-resistant bacteria is both important and challenging. The primary advantage of synergistic combinations lies in their ability to lower the concentration of each antimicrobial agent required for effective antibacterial activity [32]. This approach offers several benefits, including reduced production costs, a decreased risk of adverse side effects, diminished toxicity to mammalian cells, and a lower likelihood of drug resistance development [32]. In light of these potential advantages, we investigated the synergistic effects of BP100 and the five selected analogs in combination with ciprofloxacin against multidrug-resistant *Pseudomonas aeruginosa* (MDRPA) using a checkerboard synergy assay. Ciprofloxacin, an antibiotic belonging to the fluoroquinolone class, is commonly used to treat various bacterial infections, including urinary tract infections and pneumonia. It exerts a bactericidal effect by inhibiting bacterial DNA gyrase, thereby disrupting DNA replication and transcription and blocking protein synthesis [33]. The fractional inhibitory concentration index (FICI) results for BP100 and its selected analogs, when paired with ciprofloxacin against MDRPA, are detailed in Table 5. Notably, the combinations of BP100, BP5, BP6, BP8, BP11, and BP13 with ciprofloxacin demonstrated strong synergistic effects against MDRPA, with FICI values of 0.375, 0.3125, 0.375, 0.375, 0.3125, and 0.2656, respectively (Table 5). These findings suggest that BP100, BP5, BP6, BP8, BP11, and BP13 are promising adjuvants for use in combination with clinically employed antibiotics against antibiotic-resistant bacteria. Such combinations may offer a valuable approach to enhancing the efficacy of existing antibiotics and potentially mitigating the development of antimicrobial resistance.

## 3. Materials and Methods

### 3.1. Materials

Fresh samples of defibrinated sheep red blood cells (sRBCs) were purchased from Synergy Innovation, Seongnam, Korea. RAW264.7 (mouse macrophage) cells were purchased from the American Type Culture Collection (Manassas, VA, USA). Dulbecco’s modified Eagle’s medium (DMEM) and fetal bovine serum (FBS) were obtained from SeouLin Bioscience (Seoul, Republic of Korea). The TNF-α ELISA kit was procured from R&D Systems (Minneapolis, MN, USA). All buffers were prepared using Milli-Q ultrapure water (Merck Millipore, Burlington, MA, USA). All other reagents, including 3-(4,5-dimethylthiazol-2-yl)-2,5-diphenyl-2H-tetrazolium bromide (MTT), LPS from *E. coli* O111:B4, 3, 3′-dipropylthiadicarbocyanine iodide (diSC_3_-5), 1-N-phenylnaphthylamine (NPN), and propidium iodide (PI), were supplied by Sigma-Aldrich (St. Louis, MO, USA).

### 3.2. Bacterial Strains

*Escherichia coli* (KCTC 1682), *Pseudomonas aeruginosa* (KCTC 1637), *Salmonella typhimurium* (KCTC 1926), *Bacillus subtilis* (KCTC 3068), *Staphylococcus epidermidis* (KCTC 1917), and *Staphylococcus aureus* (KCTC 1621) were procured from the Korean Collection for Type Cultures (KCTC) of the Korea Research Institute of Bioscience and Biotechnology (KRIBB). *E. coli* ATCC 25922 was purchased from American Type Culture Collection (ATCC). Methicillin-resistant *Staphylococcus aureus* (MRSA; CCARM 3089 and CCARM 3090) and multidrug-resistant *Pseudomonas aeruginosa* (MDRPA; CCARM 2095 and CCARM 2109) were obtained from the Culture Collection of Antibiotic-Resistant Microbes (CCARM) of Seoul Women’s University in Korea.

### 3.3. Peptide Synthesis

Peptides were synthesized on rink amide MBHA resin using the Fmoc (9-fluorenylmethoxy carbonyl)-based solid-phase peptide-synthesis method. During coupling cycles, Fmoc-amino acids were added in a 10-fold excess. Piperidine was used to remove the Fmoc group. The peptides were cleaved using a TFA (trifluoroacetic acid) mixture and purified via reversed-phase preparative RP-HPLC on a Vydac C18 column with an acetonitrile gradient containing 0.05% TFA.

### 3.4. Antimicrobial Activity Assay

The minimal inhibitory concentrations (MICs) of the peptides against bacterial strains were determined using a modified broth-microdilution method in accordance with the National Committee for Clinical Laboratory Standards (NCCLS) guidelines. Overnight cultures were diluted in cation-adjusted Mueller–Hinton Broth (MHB) to achieve an inoculum density of 2 × 10^6^ CFU/mL. A two-fold serial dilution of the peptide solution (100 μL) was introduced into microtiter plates, followed by the addition of 100 μL of bacterial suspension. The plates were then incubated at 37 °C for 24 h to determine the MICs. Wells containing only culture media served as negative controls, while those with added bacteria functioned as positive controls. The MIC, expressed in μM, was defined as the lowest peptide concentration that prevented visible bacterial growth. This protocol was also employed to determine MICs in the presence of physiological salts (150 mM of NaCl, 4.5 mM of KCl, 6 μM of NH_4_Cl, 1 mM of MgCl_2_, 2.5 mM of CaCl_2_, and 4 μM of FeCl_3_) and 20% human serum to assess the peptides’ efficacy under conditions more closely resembling physiological environments. All experiments were conducted in triplicate and repeated three times independently to ensure reproducibility and statistical significance of the results.

### 3.5. Hemolytic Activity Assay

The hemolytic activity of the peptides was tested through hemolysis assay of sheep red blood cells (sRBCs). The sRBCs were diluted in PBS to achieve a 4% (*v*/*v*) concentration. Subsequently, 100 μL of peptide solutions, ranging from 1 to 256 μM, was dispensed into a 96-well plate containing an equal volume of the erythrocyte suspension. After incubating for 1 h at 37 °C and centrifuging at 1000× *g* for 10 min, 100 μL of the supernatant was transferred to a new 96-well plate. Hemoglobin release was quantified using a microplate reader at a wavelength of 450 nm. The percentage of hemolysis was calculated using percentage hemolysis = 100 × [(A_t_ − A_0_)/(A − A_0_)], where A represents the absorbance of the peptide-treated sample at 540 nm, A_0_ corresponds to the absorbance value representing 0% hemolysis in PBS, and A_t_ corresponds to the absorbance value representing 100% hemolysis in 0.1% Triton X-100.

### 3.6. Cytotoxicity Assay

The cytotoxicity of the peptides on RAW264.7 cells was evaluated using the MTT dye reduction assay. Briefly, RAW264.7 cells were seeded at a density of 2 × 10^5^ cells per well in a 96-well plate and exposed to various concentrations of the peptides for 4 h at 37 °C in a 5% CO_2_ atmosphere. Following incubation, MTT solution was added to each well, and the plates were further incubated for an additional 3 h to allow for formazan formation. The resulting formazan crystals were then solubilized using dimethyl sulfoxide (DMSO), and the absorbance was measured at 570 nm to quantify MTT reduction. Wells containing cells in culture medium without peptides served as the positive control, while wells containing only culture medium were used as the negative control.

### 3.7. Measurement of TNF-α and IL-6 Release from LPS-Stimulated RAW264.7 Cells

RAW264.7 cells were seeded in 96-well plates (5 × 10^4^ cells/well) and incubated overnight. Peptides were added, and the cultures were incubated at 37 °C for 1 h. Subsequently, 20 ng/mL LPS was added, and the cells were incubated for another 6 h at 37 °C. Release of TNF-α and IL-6 in RAW 264.7 cells was detected using the commercial ELISA kit (R&D Systems, Minneapolis, MN, USA) following the manufacturer’s protocol.

### 3.8. Biofilm Inhibition Assay (MBIC)

The inhibitory activity of the peptides on biofilm formation was evaluated against multidrug-resistant *Pseudomonas aeruginosa* (MDRPA) (CCARM 2095) using a biofilm inhibition assay to determine the minimal biofilm inhibitory concentration (MBIC). Peptide solutions were serially diluted in sterile water and aliquoted (100 μL) into a 96-well Costar polypropylene plate (Corning Inc., Corning, NY, USA). An equal volume of MDRPA suspension in Mueller–Hinton Broth (MHB), adjusted to 1 × 10^6^ CFU/mL, was added to each well, resulting in a final inoculum density of 5 × 10^5^ CFU/mL. Following overnight incubation at 37 °C, bacterial growth was quantified by measuring the optical density (OD) at 595 nm. Planktonic cells were then discarded, and the attached biofilms were washed thrice with distilled water. Residual biofilms were stained with 0.1% crystal violet for five minutes, washed to remove non-adherent dye, and subsequently destained using 95% ethanol. The OD at 595 nm was measured again to assess the biofilm mass. Control wells contained untreated bacteria. The MBIC was defined as the lowest peptide concentration that inhibited any residual crystal violet staining or biofilm formation. All assays were conducted in triplicate to ensure reproducibility. This method allows for the quantitative assessment of the peptides’ ability to prevent biofilm formation, a critical virulence factor in many bacterial infections, particularly those caused by multidrug-resistant strains.

### 3.9. Biofilm Eradication Assay (MBEC)

The minimal biofilm eradication concentration (MBEC) for peptides against the multidrug-resistant *Pseudomonas aeruginosa* (MDRPA) (CCARM 2095) strain was assessed using the Calgary Biofilm Device (CBD), supplied by Innovotech, Edmonton, AB, Canada. A bacterial suspension with a density of (1 × 10^6^) CFU/mL in (150 µL) of Luria–Bertani (LB) media was dispensed into each well of a 96-well microtiter plate, which was equipped with a peg lid (Innovotech, product code: 19111). This plate was incubated at 37 °C for 24 h with agitation at 110 rpm, promoting biofilm formation on the pegs. Subsequently, the peg lid was washed with phosphate-buffered saline (PBS, 0.01 M) and relocated to a fresh plate containing varying peptide concentrations (200 µL per well). The biofilms were then subjected to these peptides under the same incubation conditions. After another PBS rinse, the peg lid was moved to a recovery plate filled with (200 µL) of LB media per well. To detach the biofilms from the pegs, the recovery plate underwent sonication in a water bath for 10 to 15 min. Finally, the recovery plate was incubated at 37 °C for 24 h with agitation at 110 rpm, allowing any surviving bacteria to proliferate and cause turbidity. The MBEC is defined as the lowest concentration of peptides that inhibited turbidity formation in the recovery plate in comparison to sterility controls. This experiment was conducted in triplicate, and the median value from each trial was recorded.

### 3.10. Circular Dichroism (CD)

CD analysis was conducted using a Jasco-715 spectropolarimeter (Jasco, Tokyo, Japan) to assess the secondary structure of peptides in 10 mM of PBS (pH 7.4), 50% TFE, and 30 mM of SDS micelles, with the final peptide concentration at 150 µM. CD spectra were measured at a wavelength ranging from 190 to 250 nm, with a path length of 1 mm and a scanning of 100 nm/min at room temperature. Then, the mean residue ellipticity was calculated as follows: (observed ellipticity × 1000)/(peptide concentration × path length × number of amino acids).

### 3.11. Outer Membrane Permeability

The fluorescent dye NPN was used to assess the outer membrane permeability of *E. coli* (KCTC 1682). Briefly, *E. coli* cells in the mid-logarithmic phase were resuspended to an optical density at 600 nm of 0.05 in a HEPES buffer solution. This solution contained 20 mM of glucose, 5 mM of HEPES (pH 7.4), and 5 mM of KCN. Subsequently, 10 µM of NPN was added, and the background fluorescence was measured (excitation λ = 350 nm, emission λ = 420 nm). Peptides were then incrementally introduced, and the resultant changes in fluorescence were recorded.

### 3.12. Cytoplasmic Membrane Depolarization Assay

The depolarizing effect of peptides on the cytoplasmic membrane of *Staphylococcus aureus* (KCTC 1621) was evaluated using the fluorescent dye diSC_3_-5. Mid-logarithmic-phase *S. aureus* cells were harvested, washed thrice with HEPES buffer (5 mM, pH 7.4, containing 0.1 M KCl and 20 mM of glucose), and resuspended to an optical density of 0.05 at 600 nm in the same buffer. The cell suspension was then incubated with 0.4 μM of diSC_3_-5 for 90 min to allow substantial dye accumulation at the cytoplasmic membrane. To equilibrate intracellular and extracellular potassium ion (K^+^) concentrations, KCl was added to the diSC_3_-5-containing cell suspension to a final concentration of 100 mM, followed by a 15–30 min incubation at room temperature. Subsequently, peptide samples were introduced to 2 mL aliquots of the prepared suspension. Fluorescence measurements were recorded using a Shimadzu RF-5301 PC fluorescence spectrophotometer (Shimadzu, Japan) with excitation and emission wavelengths set at 622 nm and 670 nm, respectively. This assay allows for the quantitative assessment of membrane depolarization induced by the peptides, providing insights into their mechanism of action and potential antimicrobial efficacy.

### 3.13. Flow Cytometry Analysis

The damage to bacterial membrane integrity by the compounds was evaluated on *E. coli* (KCTC 1682) and *S. aureus* (KCTC 1621) membranes using an impermeable dye propidium iodide (PI) with flow cytometry. Bacteria (*E. coli* or *S. aureus*) were cultured in LB broth until they reached mid-log-phase, then washed and resuspended in PBS to a density of 2 × 10^5^ CFU/mL. The bacterial suspension was incubated with the samples and 20 µg/mL of propidium iodide (PI), a fluorescent dye, at 37 °C for 1 h in the dark. The samples were then centrifuged and washed twice in PBS to remove any excess dye. The fluorescence of PI, which indicates membrane damage, was measured by a FACS flow cytometer (Agilent, ACEA Bioscience Inc., San Diego, CA, USA) using a laser excitation wavelength of 488 nm.

### 3.14. Synergy Testing using Checkerboard Assay

The synergistic effects of peptide-antibiotic combinations against methicillin-resistant *Staphylococcus aureus* (MRSA; CCARM 3089) and multidrug-resistant *Pseudomonas aeruginosa* (MDRPA; CCARM 2095) were evaluated using the fractional inhibitory concentration index (FICI) method. Antibiotics and peptides were subjected to two-fold serial dilutions, mixed in equal ratios, and combined with a bacterial suspension adjusted to approximately 10^6^ CFU/mL. The resulting mixtures were distributed into microplate wells and incubated at 37 °C with orbital shaking at 200 rpm for 24 h. Bacterial growth was monitored by measuring the optical density at 600 nm (OD600). The FICI was calculated using the broth microdilution checkerboard method, defined as the sum of the ratios of the minimum inhibitory concentrations (MICs) of each agent in combination to their individual MICs, according to the following equation: FICI = (MIC of antibiotic in combination/MIC of antibiotic alone) + (MIC of peptide in combination/MIC of peptide alone). The interpretation of FICI values was as follows: FICI ≤ 0.5 indicates synergy; 0.5 < FICI ≤ 1.0 indicates an additive effect; 1.0 < FICI ≤ 4.0 indicates indifference; and FICI > 4.0 indicates antagonism. This method allows for the quantitative assessment of potential synergistic interactions between the peptides and conventional antibiotics, which could lead to more effective treatment strategies against multidrug-resistant pathogens.

### 3.15. Statistical Analysis

Statistical analysis was conducted using one-way analysis of variance (ANOVA) with Duncan’s multiple evaluations for comparison between more than two groups. The data of all experiments were expressed as the mean ± SD. Differences associated with *p* values of less than 0.05 were considered statistically significant.

## 4. Conclusions

In this study, we aimed to develop short α-helical multi-functional AMPs with potent antibacterial, anti-inflammatory, and antibiofilm activities while minimizing hemolytic effects. To achieve this goal, we designed and synthesized 16 Trp-substituted analogs of the cecropin A-melittin hybrid peptide BP100. Among these analogs, BP5, BP6, BP8, BP11, and BP13 demonstrated enhanced antibacterial activity against both Gram-negative and Gram-positive bacteria, including drug-resistant strains, without inducing hemolysis. Notably, these analogs exhibited a 1.5- to 5.5-fold improvement over BP100 in terms of antibacterial activity and cell selectivity. These analogs also showed superior efficacy in eradicating mature biofilms of multidrug-resistant *Pseudomonas aeruginosa* (MDRPA) compared to BP100. Furthermore, these analogs more effectively suppressed the release of pro-inflammatory cytokines in lipopolysaccharide (LPS)-stimulated RAW 264.7 cells relative to BP100. Mechanistic studies, including membrane depolarization, N-phenyl-1-naphthylamine (NPN) uptake assays, and membrane integrity assessments, revealed that BP100 and its analogs exert their antibacterial effects through a membrane-disrupting mechanism. Importantly, these BP100 analogs exhibited excellent synergistic antimicrobial activity against MDRPA when used in combination with ciprofloxacin. In conclusion, we propose that the selected BP100 analogs hold significant promise as novel antibacterial, antibiofilm, and anti-inflammatory agents. Additionally, their potential as antibiotic adjuvants for combating multidrug-resistant bacterial infections warrants further investigation. These findings contribute to the ongoing efforts to develop effective strategies against the growing threat of antimicrobial resistance and provide a foundation for future studies aimed at optimizing these peptides for potential therapeutic applications.

## Figures and Tables

**Table 1:** Amino acid sequences and physicochemical properties of BP100 and its analogs.

Peptides	Amino Acid Sequences *^a^*	Molecular Mass (Da)		Net Charge	μH *^b^*
		Calculated	Observed		
BP100	KKLFKKILKYL-NH_2_	1421.87	1420.74	+5	0.417
BP1	KKL**W**KKILKYL-NH_2_	1460.91	1459.91	+5	0.469
BP2	KKLFKK**W**LKYL-NH_2_	1494.93	1493.80	+5	0.468
BP3	KKLFKKI**W**KYL-NH_2_	1494.93	1493.77	+5	0.477
BP4	KKLFKKILK**W**L-NH_2_	1444.91	1443.87	+5	0.545
BP5 *	KK**WW**KKILKYL-NH_2_	1533.96	1532.86	+5	0.519
BP6 *	KK**W**FKKILK**W**L-NH_2_	1517.96	1516.80	+5	0.595
BP7	KKL**W**KKI**W**KYL-NH_2_	1533.96	1532.98	+5	0.519
BP8 *	KKL**W**KKILK**W**L-NH_2_	1483.95	1482.94	+5	0.586
BP9	KKLFKK**WW**KYL-NH_2_	1567.98	1566.80	+5	0.518
BP10	KKLFKK**W**LKY**W**-NH_2_	1567.98	1566.78	+5	0.518
BP11 *	KKLFKKI**W**K**W**L-NH_2_	1517.96	1516.99	+5	0.595
BP12	KKLFKKILK**WW**-NH_2_	1517.96	1516.96	+5	0.595
BP13 *	KK**W**FKK**W**LK**W**L-NH_2_	1591.02	1589.89	+5	0.635
BP14	KKL**W**KK**W**LKY**W**-NH_2_	1607.02	1605.80	+5	0.560
BP15	KKL**W**KKI**W**KY**W**-NH_2_	1607.02	1605.82	+5	0.569
BP16	KKLFKK**WW**K**W**L-NH_2_	1591.02	1589.69	+5	0.635

*^a^* Bold characters are the amino acid residues that were substituted in this study. *^b^* Mean hydrophobic moment (μH) calculated from HeliQuest tool. * Represents the peptides chosen as the ideal candidate for further analysis.

**Table 2:** Antimicrobial activities of BP100 and its analogs against bacterial strains.

Bacterial Strains	Minimal Inhibitory Concentration (MIC) *^a^* (μM)																	
	BP100	BP1	BP2	BP3	BP4	BP5	BP6	BP7	BP8	BP9	BP10	BP11	BP12	BP13	BP14	BP15	BP16	ME
Gram-positive bacteria																		
*S. aureus* (KCTC 1621)	4	8	4	8	4	2	2	2	4	8	8	4	4	4	2	2	16	8
*S. epidermidis* (KCTC 1917)	4	8	2	8	8	1	2	2	2	4	8	4	4	8	2	4	16	32
*B. subtilis* (KCTC 3068)	8	8	8	16	4	2	4	4	8	16	16	8	8	8	4	4	16	16
Resistant Gram-positive bacteria																		
MRSA *^b^* (CCARM 3089)	32	32	16	64	64	4	8	8	16	64	64	8	64	32	16	16	16	32
MRSA (CCARM 3090)	64	64	16	64	64	8	16	32	16	64	64	32	64	32	32	64	128	8
VREF *^c^* (ATCC 51559)	64	64	128	64	16	8	8	32	16	64	128	16	128	16	64	64	64	64
Gram-negative bacteria																		
*E. coli* (KCTC 1682)	4	8	2	8	4	2	4	4	2	4	8	8	8	8	4	4	16	8
*P. aeruginosa* (KCTC 1637)	16	16	8	32	16	4	16	8	16	16	8	8	16	16	8	8	8	16
*S. typhimurium* (KCTC 1926)	4	8	8	4	2	2	4	2	4	4	8	2	4	8	8	4	16	16
Resistant Gram-negative bacteria																		
MDRPA *^d^* (CCARM 2095)	32	16	8	16	8	8	8	8	8	16	16	8	16	8	16	16	32	32
MDRPA (CCARM 2109)	16	16	4	16	16	4	16	4	16	16	16	16	16	16	8	8	32	32
GM *^e^*	22.5	22.5	18.5	27.3	18.7	4.1	8.0	9.6	9.8	25.1	31.3	10.4	30.2	14.2	14.9	17.6	32.7	24.0
HC_10_ *^f^*	>256	>256	>256	>256	>256	>256	>256	>256	>256	>256	>256	>256	>256	>256	>256	>256	>256	2.0
TI *^g^*	22.7	22.7	27.7	18.8	27.4	124.9	64.0	53.3	52.2	20.4	16.4	49.2	17.0	36.1	34.4	29.1	15.7	0.08

ME: melittin; *^a^* MIC was determined as the lowest concentration of peptide that caused 100% inhibition of microbial growth; *^b^* MRSA: methicillin-resistant *Staphylococcus aureus*; *^c^* VREF: vancomycin-resistant *Enterococcus faecium*; *^d^* MDRPA: multidrug-resistant *Pseudomonas aeruginosa*; *^e^* GM denotes the geometric mean of MIC values from selected bacterial and fungal strains; *^f^* HC_10_ is the peptide concentration that caused 10% hemolysis of sheep red blood cells (sRBCs); *^g^* therapeutic index (TI) is the ratio of the HC_10_ value (μM) over GM (μM). When no detectable hemolytic activity was observed at 256 μM, a value of 512 μM was used to calculate the TI.

**Table 3:** Antibiofilm activity of BP100 and its analogs against MDRPA planktonic bacteria and biofilms.

Peptides	MBIC_50_ (μM)/MBIC_90_ (μM)	MBEC_50_ (μM)/MBEC_90_ (μM)
BP100	4–8/8	16–32/32
BP1	4–8/8	8/16
BP2	8–16/16	16–32/32
BP3	8–16/16	16/32
BP4	4–8/8	8/32
BP5	4–8/8	2/8
BP6	4–8/8	4/8
BP7	4–8/8	4–8/8
BP8	4–8/8	2/8
BP9	8–16/16	4/8
BP10	16–32/32	8–16/16
BP11	4–8/8	4–8/8
BP12	4–8/8	8–16/16
BP13	4–8/8	4/8
BP14	8–16/16	8–16/16
BP15	4–8/8	8–16/16
BP16	8–16/16	16/32
LL-37	32–64/64	32/>64

MBIC: minimum biofilm inhibition concentration; MBEC: minimum biofilm eradication concentration.

**Table 4:** The MIC values (μM) of the peptides in the presence of physiological salts and human serum against *E. coli* (KCTC 1682) and *S. aureus* (KCTC 1621).

Peptides	Control	150 mM of NaCl	4.5 mM of KCl	6 μM of NH_4_Cl	1 mM of MgCl_2_	2.5 mM of CaCl_2_	4 μM of FeCl_3_	10% Human Serum
*E. coli* (KCTC 1682)								
BP100	4	16	16	16	16	16	16	16
BP5	2	8	4	4	4	8	8	4
BP6	4	8	4	16	8	16	16	4
BP8	2	8	4	8	8	16	4	4
BP11	8	16	8	4	8	16	4	8
BP13	8	16	8	8	8	16	8	16
*S. aureus* (KCTC 1621)								
BP100	4	16	16	16	16	16	16	32
BP5	2	4	4	4	4	4	4	4
BP6	2	4	4	4	4	4	8	4
BP8	4	4	4	4	4	4	4	4
BP11	4	8	4	4	4	16	4	8
BP13	4	8	8	8	8	8	8	8

The control MICs were determined in the absence of salts.

**Table 5:** Synergistic antimicrobial activity of the peptide with ciprofloxacin (CIP) against MDRPA (CCARM 2095).

Peptides	MIC_A_	[A]	FIC_A_	MIC_B_	[B]	FIC_B_	FICI*^a^*	Interpretation
BP100	32	4	0.125	2048	512	0.25	0.375	synergy
BP5	8	0.5	0.0625	2048	512	0.25	0.3125	synergy
BP6	8	1	0.125	2048	512	0.25	0.375	synergy
BP8	8	0.5	0.0625	2048	512	0.25	0.375	synergy
BP11	8	0.5	0.0625	2048	512	0.25	0.3125	synergy
BP13	8	0.125	0.015625	2048	512	0.25	0.2656	synergy

MIC: Minimal inhibitory concentration; MIC_A_: MIC (μg/mL) of peptide alone; [A]: MIC (μg/mL) of peptide in combination; MIC_B_: MIC (μg/mL) of ciprofloxacin alone; [B]: MIC (μg/mL) of ciprofloxacin in combination; FIC_A_: fractional inhibitory concentration of peptide; FIC_B_: fractional inhibitory concentration of ciprofloxacin; FICI, fractional inhibitory concentration index; *^a^* FICI: [A]/MIC_A_ + [B]/MIC_B_; FICI ≤ 0.5 was interpreted as synergy; 0.5 < FICI ≤ 1.0 as additive; 1.0 < FICI ≤ 4.0, as indifferent; and FICI > 4.0 as antagonism.

## Data Availability

Data are contained within the article and the Supplementary Materials.

## Supplementary Materials

The following supporting information can be downloaded at https://www.mdpi.com/article/10.3390/molecules29225231/s1. Figure S1: MADI-TOF-MS of synthetic BP100 and its analogs. Figure S2: The cytotoxicity of BP100 and its analogs against mouse macrophage RAW264.7 cells.
