# Supplementary material for: Enhanced Antibacterial, Anti-Inflammatory, and Antibiofilm Activities of Tryptophan-Substituted Peptides Derived from Cecropin A-Melittin Hybrid Peptide BP100
Source: Molecules. 2024 Nov 5;29(22):5231. doi: 10.3390/molecules29225231 (PMC11596392; doi:10.3390/molecules29225231)
Supplement: Supplementary file 1 [file molecules-29-05231-s001.zip › molecules-3268480-supplementary.pdf]

Supplementary Materials

Enhanced Antibacterial, Anti-inflammatory, and Antibiofilm Activities of Tryptophan-Substituted Peptides Derived from Cecropin A-Melittin Hybrid Peptide BP100

Sukumar Dinesh Kumar [1,†,] Eun Young Kim [1,†,] Naveen Kumar Radhakrishnan [2,†,] Jeong Kyu Bang [3,†,] Sungtae Yang [4,5] and Song Yub Shin [1,2,*]

[1] Department of Cellular & Molecular Medicine, School of Medicine, Chosun University, Gwangju 61452, Republic of Korea; sdkumarphd@gmail.com (S.D.K.); lovetive@naver.com (E.Y.K.)

[2] Graduate School of Biomedical Science, Chosun University, Gwangju 61452, Republic of Korea; naveens4596@gmail.com

[3] Division of Magnetic Resonance, Korea Basic Science Institute (KBSI), Ochang 28119, Republic of Korea; bangjk@kbsi.re.kr

[4] Departme nt of Microbiology, School of Medicine, Chosun University, Gwangju 61452, Republic of Korea; styang@chosun.ac.kr

[5] Institute of Well-Aging Medicare & CSU G-LAMP Project Group, Chosun University, Gwangju 61452, Republic of Korea; styang@chosun.ac.kr

* Correspondence: syshin@chosun.ac.kr

† These authors contributed equally to this work..

Figure S1. MALDI-TOF-MS of synthetic BP100 and its analogs

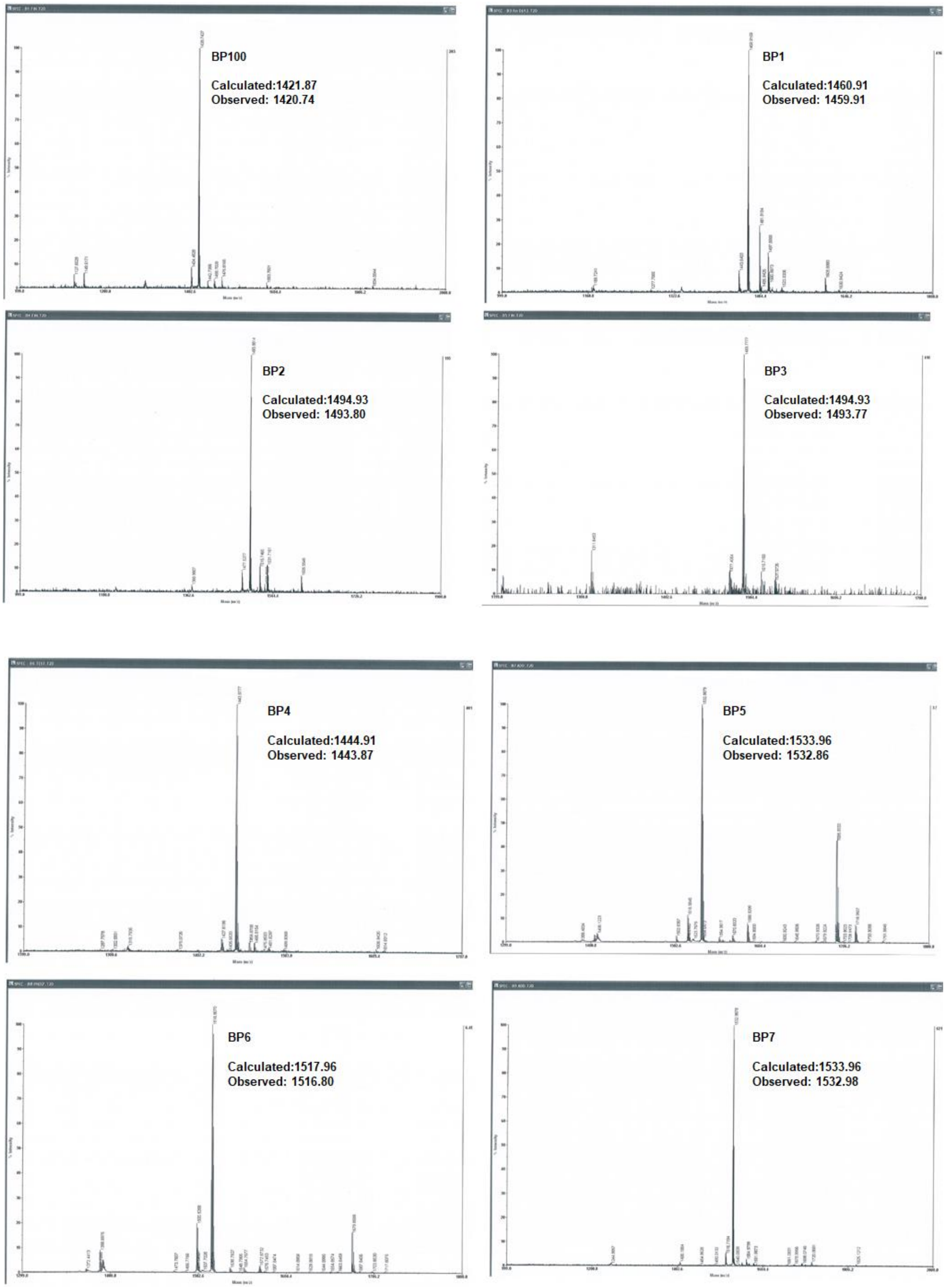

Figure S1. Continued

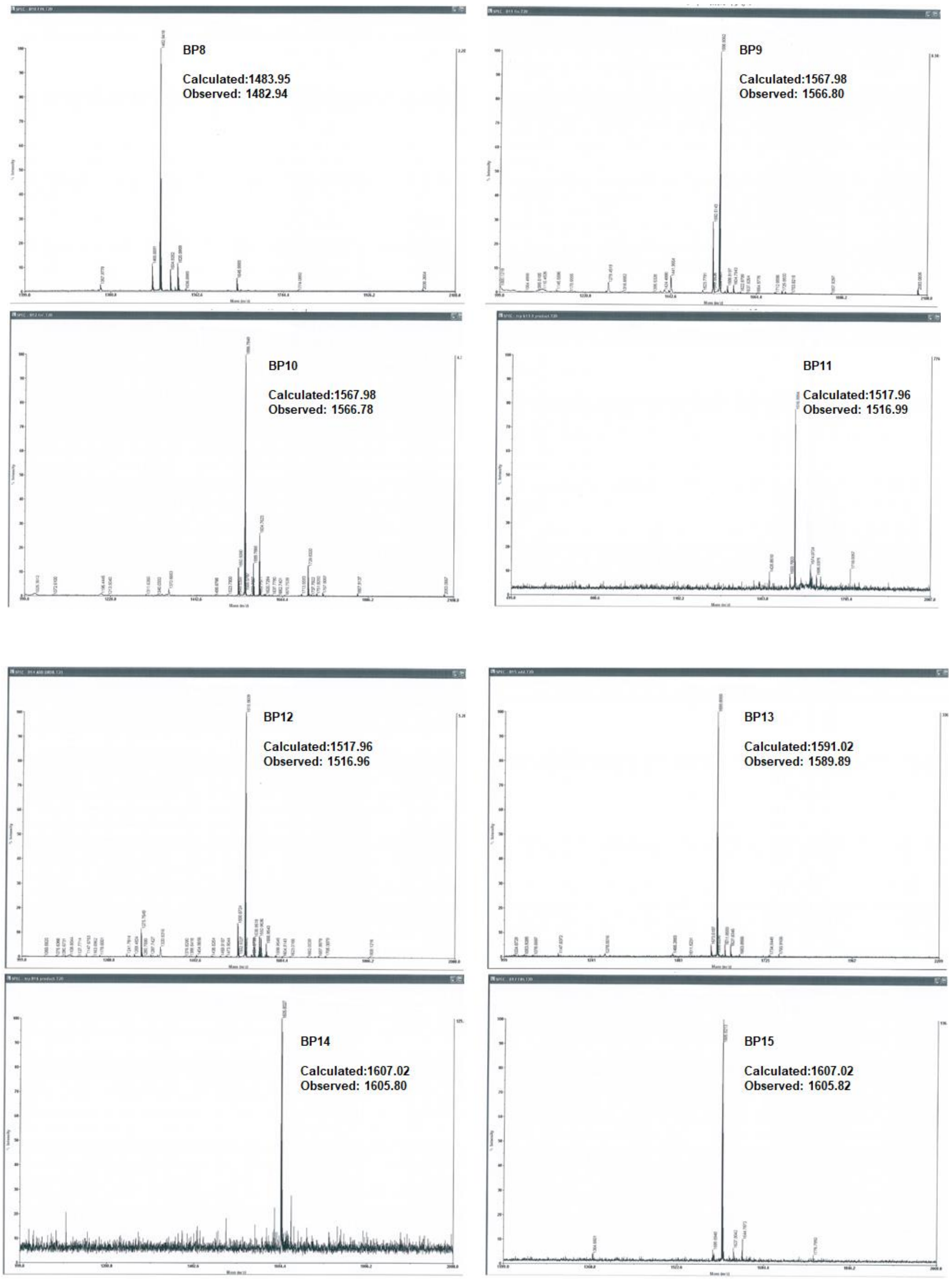

Figure S1. Continued

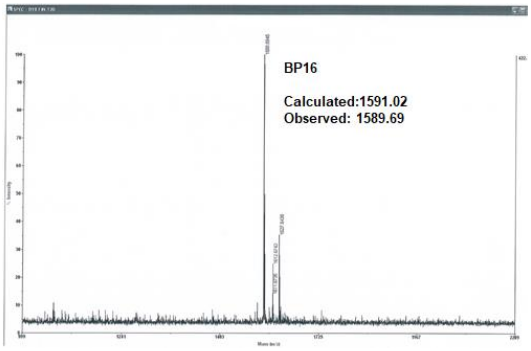

Figure S2. Cytotoxicity of BP100 and its analogs against mouse macrophage RAW264.7 cells

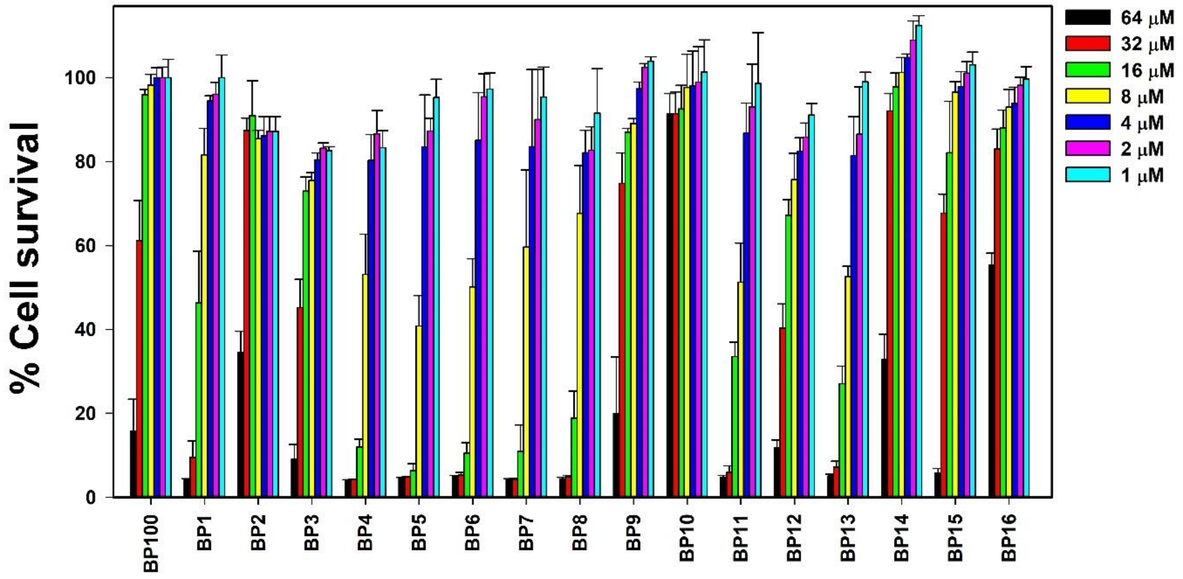

Figure S2. Cytotoxicity of BP100 and its analogs against RAW264.7 macrophages assessed by MTT assay. Cells were treated with varying concentrations (1-64 μM) of peptides for 24 h. Cell viability is expressed as a percentage relative to PBS-treated cells (negative control, defined as 100% survival) and Triton X-100-treated cells (positive control, 0% survival). Data represent mean ± SD from three independent experiments performed in triplicate.
